# Revisiting the Explanations of the Beta-Sheet Twist and Its Handedness

**DOI:** 10.3390/ijms27041899

**Published:** 2026-02-16

**Authors:** Beatrice Ruth, Maximilian Fichtner, Stefan Schuster

**Affiliations:** 1 Biosystems Analysis Group, Faculty of Mathematics and Computer Science, Friedrich Schiller University Jena, Inselplatz 5, 07743 Jena, Germany; beatrice.flick@uni-jena.de; 2 Department of Bioinformatics, Faculty of Biological Sciences, Friedrich Schiller University Jena, Inselplatz 5, 07743 Jena, Germany

**Keywords:** *β*-sheet twist, steric hindrance, Ramachandran plot, handedness, dihedral angles, intrastrand interactions

## Abstract

The β-sheet, consisting of several β-strands, is one of the most important secondary structures of proteins. Most β-sheets differ greatly from the fully extended, all-trans form due to twisting and/or bending. When looked at in the direction of the β-strands rather than along the hydrogen bonds, the twist is usually right-handed. Although numerous studies have investigated the origin of the right-handed twist of β-sheets or β-strands in proteins, there is no common agreement about its causes. The twist can be seen from the dihedral angles in the Ramachandran plot. Here, we discuss the opposing roles of the dihedral angles ϕ and ψ. The key role is played by the angle ϕ, which is controlling the distance between the carbonyl group of the backbone and the side chain of the next amino acid. There are two antisymmetric effects: the change in ϕ in the clockwise direction is initiated by a $C_{\beta }$… O clash and delimited by a subsequent $C_{\beta }$… NH clash, while the opposite relationship holds for the counter-clockwise change in ψ. The impact of the twist on tertiary structures is examined. The understanding of the molecular effects within a strand is deepened by 3D computer images and ball–and–stick models. The use of (tangible) physical models is highlighted in view of teaching structural biology to undergraduate students.

## 1. Introduction

### 1.1. Background

**Figure 1:**
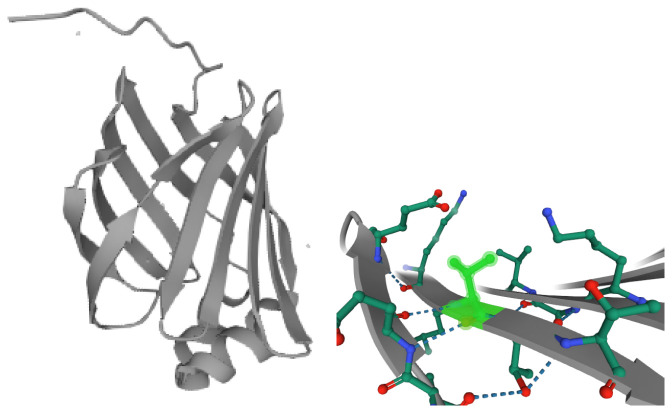
Three-dimensional structure of the fatty acid-binding protein localized in human liver (UniprotID: P07148, PDB ID: 2F73). (**Left**) Entire protein, with the tertiary structure of a flattened β-barrel. (**Right**) Zoomed in on valine 114, showing the twist of neighboring strands.

Secondary structures of proteins are the subject of intensive research due to their relevance for manifold applications [1,2,3,4,5,6]. An important secondary structure is the β-sheet, described by Pauling and Corey in 1951 [7], consisting of several β-strands that are mainly connected to each other via hydrogen bonds between neighboring strands (Figure 1) [8,9,10]. The β-strands can be arranged in a parallel, antiparallel, or mixed way [8,9,10].

**Figure 2:**
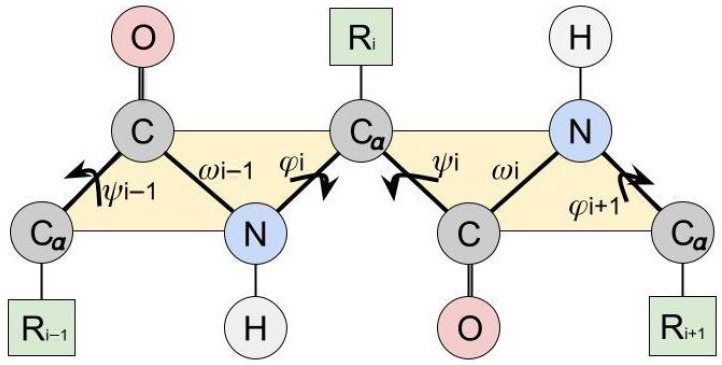
Schematic representation of dihedral angles. A small section of the protein backbone is displayed in atomic detail, with the dihedral angles ϕ, ω, and ψ. The partial double bond nature of the peptide bond gives rise to a planar configuration of the carbonyl group (CO), amino group (NH), and their neighboring $C_{\alpha }$ atoms. Standard atom coloring applied; residues shown in green. Arrows illustrate the torsion angles.

In the fully extended β-sheet, all dihedral angles ψ, ω, and ϕ are equal to 180° and, thus, are in the trans conformation. Most β-sheets differ considerably from this fully extended form due to twisting and/or bending, implying that the dihedral angles ψ and ϕ are unequal to 180° [3,4]. To distinguish twisting from bending, four neighboring amino acids within a β-strand have to be examined, while for analyzing the twist, three amino acids may be sufficient, as explained below. Through their peptide bonds, limiting ω to 180°, directly neighboring $C_{\alpha }$ atoms of the backbone ideally form a flat plane (see Figure 2). There is a twist if the next peptide bond is outside this plane [4,11]. There is a bend if there is no straight line between the midpoints of three neighboring peptide bonds [4,11].

The twist and bend of β-sheets are important structural aspects as they allow the hierarchical formation and aggregation of multiple beta-sheets to form tertiary structures in the form of, for example, β-barrels, propellers, and silk fibroin, which include antiparallel β-sheets [4,5,12]. The resulting cavities in β-sheets also facilitate the binding of small molecules [6,13]. In this paper, we focus on the twist rather than the bend.

The β-sheet twist is characterized by chirality. To define its handedness, the direction of viewing needs to be decided upon. Despite considerable variations in both the length and number of individual β-strands, virtually all β-sheets, viewed in the direction of the strands, show a right-handed twist [1,11,14,15,16]. The geometrical properties of planes then imply that the twist is left-handed when viewed perpendicularly to the strands. As an exception, a left-handed twist (in the direction of strands) can be realized artificially by enzyme digestion and re-precipitation of *Bombyx mori* silk [17]. Polyglycine sheets are not twisted for two reasons: glycine is achiral and the only atom in its side chain is a hydrogen atom [14,18]. Thus, less steric hindrance should be expected.

Numerous explanations for the β-sheet twist have been proposed previously. These can be categorized into two types: thermodynamic and molecular explanations. The former revolve around an argument in terms of an increase in entropy according to the second law of thermodynamics [15]. These two types of reasoning do not exclude each other; they start from different viewpoints. These explanations, which will be detailed in Section 1.2, are, however, neither detailed nor very clear and have not entered textbook knowledge so far. They remain controversial and the phenomenon is still not fully understood. An important question is whether the β-sheet twist is mainly caused by intrastrand or interstrand interactions [3,4,5,19,20].

Here, we revisit and evaluate the explanations of the β-sheet twist and its handedness proposed in the literature and extend one of them. In particular, we shed light on the different roles of the ψ and ϕ angles within a single strand. We exemplify the study by computer simulations using the Avogadro software and visualize it by photographs of plastic molecule models. The use of these models is discussed in view of teaching structural biology to undergraduate students.

### 1.2. Structural Biology and Historical Perspective

**Figure 3:**
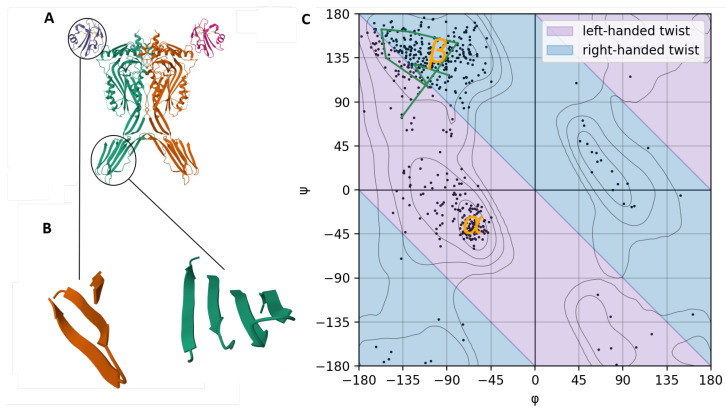
Spatial structure and Ramachandran plot of the toxin receptor complex with intermedilysin complexed to CD59 according to PDB (5IMT in that database). (**A**) Ribbon model of that protein complex, consisting of α-helices and β-sheets. (**B**) View of a part of that protein complex zoomed in, showing the twist. Green: Intermedilysin residues 419–427, 465–473 and 508–527. Orange: Glycoprotein CD59 residues 24–39 and 61–64. (**C**) Ramachandran plot of the toxin receptor complex displaying all dihedral angles of the backbone using the ramachandraw software (version 1.0.1) [21]. Greek letters (in orange) correspond to secondary structure elements. Light blue background, right-handed twist; light purple background, left-handed twist [15]. The green track connects the points corresponding to intermedilysin residues 519–526 in the right-most strand in the β-strand colored in green in (**B**). For further explanation, see text.

Two examples of proteins with a β-sheet twist are shown in Figure 1 and Figure 3. Neighboring strands point in slightly different directions. It can be seen that the twist is right-handed in the direction of the strands.

Each dihedral angle is determined by four atoms, notably the two atoms forming the bond for which the angle is defined and two adjacent atoms, which can rotate around the axis of that bond (see Figure 2). The latter two atoms have maximum distance if the dihedral angle equals 180°. Applying this reasoning to the entire chain, it can be derived that the protein chain reaches its maximum length if all dihedral angles equal 180°.

This fully extended, all-trans conformation is highly ordered and, thus, has a low entropy. In other words, the macrostate of that conformation only corresponds to one microstate with respect to dihedral angles, notably that all are equal to 180°. Applying Boltzmann’s formula S=k log *W* for entropy (with *k* and *W* standing for the Boltzmann constant and the number of microstates, respectively), we see that the fully extended conformation implies S=0 (neglecting other contributions to entropy). In twisted conformations, entropy can be higher because an observed macrostate with a given overall twist can correspond to many microstates with different possible compositions of values of dihedral angles. However, it is worth noting that while a twist can only occur if the chain is not fully extended, the converse conclusion does not hold: A contracted chain need not show a twist, for example, if it corresponds to points lying on the diagonal line in the Ramachandran plot; see Section 4.1. In fact, for symmetry reasons, the entropy argument does not favor a right-handed nor left-handed twist.

Chothia [15] used the following argument in terms of entropy. Since the admissible region above the diagonal in the Ramachandran plot is larger than the region below it, the entropy of residing in the former region is larger. In our opinion, a purely thermodynamic argument is not completely convincing because it does not explain why the upper region is larger. Moreover, it is unlikely that a β-sheet is flexible enough to cover the entire upper region by its molecular vibrations. A more profound molecular explanation is needed. In fact, Chothia mentioned, in the same paper [15], steric hindrance between the carbonyl oxygen in the backbone and the side chain of the next amino acid, as will be explained in detail below.

A second explanation is based on the steric hindrance between amino acid side chains. Since neighboring residues point to opposite directions, they do not hinder each other. In contrast, the second-next side chains point to the same direction. With a moderate twist, hindrance is reduced because the side chains are shifted almost perpendicular to the axial repeat and, thus, their distance is increased [22]. However, if the twist gets too large, the side chains come nearer to each other again because the protein backbone is contracted and, in addition, other atoms may cause a hindrance. Steric effects between second-next residues are low because of the large distance, notably the axial repeat of about 6.5–7.0 Å [22]. Importantly, the steric hindrance of second-next side chains cannot explain the handedness of the twist because clockwise and counter-clockwise twists reduce the steric hindrance explained above to the same extent [22].

To explain the chirality of the twist, it is more suitable to consider steric hindrance between side chains and the backbone within the same or of a neighboring amino acid [3,15,22]. As mentioned above, Chothia (1973) discussed the steric hindrance between the carbonyl oxygen in the backbone and the side chain of the next amino acid [15]. Alleviating that hindrance indeed leads to the correct handedness. An O … $C_{\beta }$ clash was also discussed by Ho and Curmi (2002) [3]. However, they apparently refer to another clash than Chothia [15], notably the hindrance between atoms within the same amino acid. Accordingly, they studied its dependence on the ψ angle. Moreover, they argued that this cannot be the only cause and that other factors need to be considered, such as the so-called bifurcated H-bonds (see further below in this subsection).

Chou and Scheraga [22] proposed that in addition to interactions between second-next side chains, also $C_{\gamma }$… O and $C_{\gamma }$… NH clashes contribute to the phenomenon and make the right-handed twist energetically more favorable. Further molecular explanations include electrostatic attraction between the partial charge of the carbonyl carbon and that of the carbonyl oxygen of an adjacent residue [23] and an eclipsed conformation of the lone electron pair of nitrogen and the $C_{\alpha }$-carbonyl C bond [24]. However, the latter reasoning can be questioned because the peptide bond has a partial double bond character. Therefore, two of the outer electrons of the N atom are located in the p orbital and one in the three ${\mathrm{sp}}^{2}$ orbitals each. Thus, the N atom does not include a lone electron pair. Reviews on the potential causes of the twist were presented by Ho and Curmi [3] and by Koh and Kim [19].

Interstrand interactions are due to hydrogen bonds and various steric effects involving side chains and possibly the backbones between neighboring strands. Also about these, very different views can be found in the literature. Some authors consider these interactions to be the main driving force for the twist [18]. Others stated that intrastrand interactions would be more important [15,22,24], with some of them even arguing that interstrand interactions favor flat structures [8,22]. Still others consider both types to be important [3,14,20,25].

If hydrogen bonds are formed only between carbonyl and NH groups, they should not favor any twist because the bond angles in these groups are 120°, so that the backbone of adjacent chains and the hydrogen bonds between them can lie in one plane (Figure 2) [19]. Some authors advocate the existence of bifurcated hydrogen bonds with carbonyl oxygens being the acceptors. The donors are NH groups in a neighboring strand (as proposed earlier by Pauling and Corey [7]) and the $C_{\alpha }$H group in that neighboring strand. Such $C_{\alpha }$H-O bonds correspond to a slightly different structure of the β-sheet proposed by Fraser and MacRae (1973) [26]. This model appears to explain the microstructure of some fibrous proteins better than the Pauling model. Moreover, bifurcated H-bonds may stabilize the twist [3].

An explanation related to interstrand interactions is that β-barrels would form minimal surfaces [19]. However, that would imply that their diameter would be lower in the middle than at their ends. Chou and coworkers (1983) stated that the right-handed twist is a result of intrachain interactions in the case of poly-L-valine, while these interactions would favor a left-handed twist in poly-L-isoleucine, and the right-handed twist in the latter would be the result of interchain interactions [20]. An important factor is the steric hindrance between residues of neighboring strands [14,20,25]. This is particularly relevant in antiparallel β-sheets because therein, every second residue is opposing a residue in the neighboring strand.

## 2. Results

### 2.1. Conclusions Derived from the Ramachandran Plot

The region for β-sheets lies mostly within the upper left quadrant of the Ramachandran plot with small regions within the lower left quadrant and lower right quadrant with ϕ ranging from 160° surpassing ±180° to −70° and ψ ranging from −160° over ±180° to 80° (Figure 3C).

The beta strand reaches its maximum length in the point (±180°, ±180°), any other angle combination leads to a contraction of the strand. It can be seen from the Ramachandran plot that within the region of β-sheets, ϕ is somewhat more flexible than ψ.

As mentioned in Section 4.1, the subregion above the diagonal line corresponds to right-handedness (Figure 3C, light-blue area). This subregion, mostly located within the upper left quadrant of the plot, also stretches out to the lower left and lower right quadrant of the plot [15]. To enter this subregion in the upper left quadrant from an all-trans conformation, ϕ has to be increased more (rotated clockwise) than ψ is decreased (rotated counter-clockwise). To reach the lower left quadrant, ψ is also increased, thus contributing to the right-handed twist as well. In the lower right quadrant, ϕ has to be less decreased than ψ is increased, leading to an overall locally right-handed twist. It is rare, though, that ψ is contributing to a right-handed twist (Figure 3).

The second subregion is below the diagonal line and indicates a locally left-handed twist (Figure 3C, light-purple area). Within this subregion, most of the points belonging to β-sheets are located in the upper left quadrant. Still there are also rare combinations in the lower right quadrant of the plot (Figure 3).

As the β-sheet region is mostly located in the upper left quadrant of the Ramachandran plot and has its center above the diagonal line, most local twists are right-handed. Note that each point corresponds to one amino acid. A point below the diagonal does not imply that an entire β-strand would have a left-handed twist; it is usually over-compensated by many points of the same strand above the diagonal. This is exemplified by the green track in Figure 3C.

Although the Ramachandran plot is very useful for illustrating spatial structures such as the β-sheet twist, it is only a phenomenological description and does not provide a mechanistic explanation. To give an explanation of the β-sheet twist and its handedness, we reveal the contributing factors in the following subsections.

### 2.2. Simulations with Avogadro

**Figure 4:**
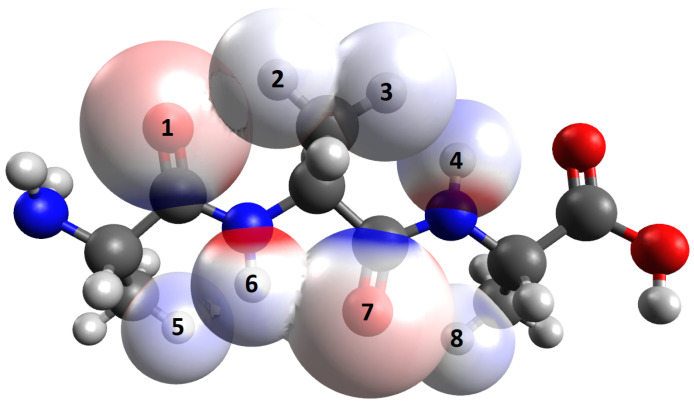
Structure of an all-trans alanine tripeptide as computed by Avogadro. Using that software, interatomic distances between O (1) and H (2) (on $C_{\beta }$), and between O (7) and H (8), were determined as 2.4 Å and those between H (3) and H (4), and between H (5) and H (6) as 2.3 Å. The overlap of the Bondi van der Waals radii (O, 1.5 Å; H, 1.2 Å) implies that these distances are slightly compressed, albeit with marginal steric repulsion for the hydrogen pairs H (3)/H (4) and H (5)/H (6).

**Figure 5:**
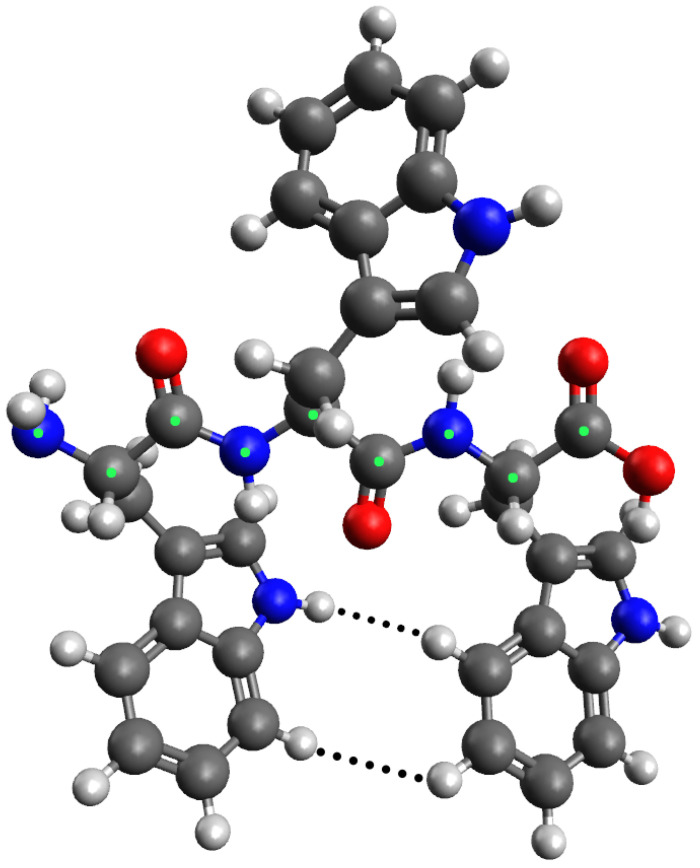
Structure of an all-trans tryptophan tripeptide as computed by Avogadro. Green dots highlight carbons and nitrogens in the backbone, distinguishing them from the side chains. The carbonyl oxygen atoms hinder hydrogens of the $C_{\beta }$ atoms in the next amino acid sterically. This can be reduced by increasing the ϕ angles. Steric hindrance of the first and third side chains plays a role as well. The distance between the two hydrogen atoms (dotted lines), which are the closest to each other in the side chains, amounts to 3.4 Å (upper line) and 3.2 Å (lower line). For comparison, the axial repeat is between 6.5 Å and 7.0 Å in usual β strands.

As an illustrative example, a tri-alanine peptide was simulated using Avogadro [27] (Figure 4). Even though alanine is not very common in β-sheets [28] except in silk fibroin [17], it was chosen because of its small size, which simplifies the graphical representation. Peptides made up solely of alanine have been used as model systems for β-sheets earlier [14]. The side chain effects contributing to the observed dihedral angles in β-sheets can nicely be illustrated by alanine in spite of its smallness. For comparison, we also studied a tri-tryptophan peptide (Figure 5).

As a single β-strand is here modeled by a tripeptide only, the N- and C-terminal effects also had to be considered. Another study about the increased stability of antiparallel versus parallel sheets showed that edge effects may change the total amount of twist but the handedness will remain the same [14].

Without any twist, a straight chain of three amino acids in all-trans conformation shows the fully elongated form (Figure 4). The Bondi van der Waals radii of hydrogen and oxygen amount to 1.2 Å and 1.5 Å, respectively [29]. Avogadro’s integrated distance measurement was used to compute the distances between the atoms of the backbone and the side chain atoms. It turned out that the distance between the oxygen atom and the side chain hydrogen atom of the following amino acid of 2.4 Å in the fully extended form is smaller than the required distance of the sum, 2.7 Å, of the two above-mentioned radii.

**Figure 6:**
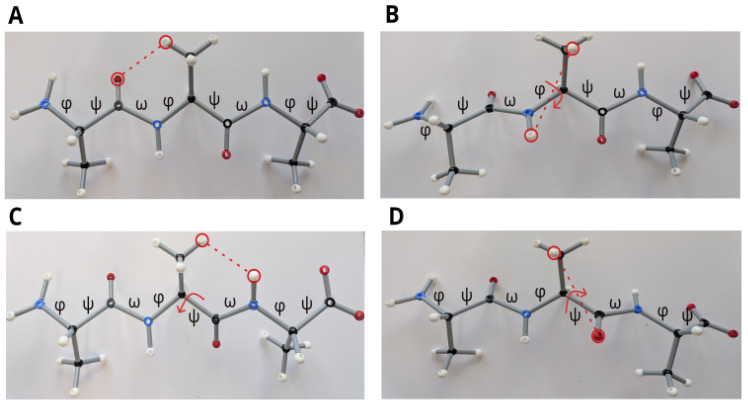
Ball–and–stick model of an alanine tripeptide constructed with an ’ORBIT’ molecule model kit [30]. Standard atom coloring is applied; distances within several pairs of atoms are indicated by red circles and dotted lines. (**A**) All-trans conformation. The strongest steric hindrance is between the oxygens and the next (in the direction towards the C-terminus) side chains, notably about 6 cm in the model, which corresponds to ≈2.6–3.0 Å according to the conversion table of the above-mentioned kit. (**B**) Conformation with ϕ of the amino acid in the middle changed clockwise from ±180° to −90° (red arrow). The distance between the oxygen of the amino acid on the left and the side chain of the amino acid in the middle has increased. However, the distance between the amino hydrogen and the side chain of the same amino acid in the middle decreases upon this clockwise rotation of ϕ (red dotted line), so that a compromise needs to be found. (**C**) Conformation with ψ of the amino acid in the middle changed clockwise from ±180° to −165° (red arrow). The amino hydrogen is now nearer to the side chain atoms of the previous amino acid than in the all trans-conformation. (**D**) Conformation with ψ of the amino acid in the middle changed anticlockwise from ±180° to 120° (red arrow). While the distance of the amino hydrogen to the side chain atoms of the previous amino acid has increased in comparison to the all-trans conformation, the oxygen of the amino acid in the middle is now closer to the side chain of the same amino acid (red dotted line).

The distance between these atoms can be increased by a twist in the opposing bond, which corresponds to a change in ϕ. Figure 6 shows that in the direction from the N- to the C-terminus, ϕ can only be changed clockwise because in the other direction, the side chain would come even closer to the carbonyl oxygen atom of the backbone. Therefore, the small distance between these atoms is crucial for inducing a right-handed twist. The distance between the backbone amino hydrogen and the nearest side chain hydrogen is 2.3 Å (Figure 6). Their Bondi van der Waals radii add up to 2.4 Å. So, due to the short side chain, only minimal or no changes to ψ may be required, given the bonds present in the model of the tri-alanine peptide. A ball–and–stick model makes it easier to understand the effects of changing ψ; see next subsection.

In contrast to alanine, particularly common in β-sheets are the amino acids valine, isoleucine, phenylalanine, tyrosine, cysteine, and tryptophan [28]. By way of example and to study the extreme case of the largest proteinogenic amino acid, we here consider a tri-tryptophan peptide. In Figure 5, a simulation of the spatial structure of that peptide in all-trans conformation is shown. It can be seen that the $C_{\beta }$ and the atoms in the side chain connected to it sterically hinder the carbonyl oxygen in the previous amino acid (when looking from the N- to the C-terminus). This can be reduced by increasing the ϕ angle. This demonstrates that the twist is not only relevant in the case of small residues like alanine but also for large amino acids.

In Figure 5, it can be seen that in the case of voluminous amino acids, the steric hindrance of second-next side chains should also be considered. This corresponds to an explanation for the twist proposed earlier [22]. However, the steric hindrance between atoms in the backbone and in side chains is more important because the distances are shorter than between second-next side chains. This reasoning is supported by chirality arguments. At the molecular level, the handedness is caused by the chirality of the amino acids involved. In interactions between second-next side chains, two L-amino acids cause the same effect as two D-amino acids (because of the rule of signs saying that both minus times minus and plus times plus give plus). In contrast, in the interaction between one amino acid and the backbone, chirality does matter.

### 2.3. Analysis via Ball–and–Stick Models

Observations on physical (rather than in silico) ball–and–stick models serve to facilitate the general understanding of the spatial structure (Figure 6). Various conformations can be tried out manually. Thus, we can elaborate on the reasoning in the previous subsection in more detail.

Two different $C_{\beta }$… O clashes have to be taken into account (Figure 2). One of these occurs in and near the all-trans conformation between the side chain (in particular, the $C_{\beta }$ and atoms in the side chain connected to it) and the carbonyl group of the previous amino acid (Figure 6A). The other becomes relevant in the twisted conformation between the side chain and the carbonyl group within the same amino acid (Figure 6D). The former clash can be reduced by clockwise rotation of ϕ. Note that a rotation of the dihedral angle $\chi_{1}$ in the side chain may alleviate the clash to a very small extent only and the dihedral angle ω, which is situated in between as well, is fixed. Upon a clockwise rotation of ϕ, the side chain approaches the NH group of the same amino acid (Figure 6B). This steric hindrance is the limiting factor for that rotation.

The change in ψ is initiated by the steric hindrance between the side chain and the NH group of the next amino acid (Figure 6C), which is attenuated by counter-clockwise rotation. Here, the limiting factor is the approach of the side chain and carbonyl O in the same amino acid (Figure 6D). Thus, the two effects are antisymmetric to each other: the change in ϕ in the clockwise direction is initiated by the clash between the side chain and a CO and delimited by the subsequent clash of the side chain and an NH, while it is the other way around for the counter-clockwise change in ψ. The overall twist depends on which rotation predominates. This question is easy to answer because the CO group is larger than the NH group, so that the initial $C_{\beta }\ldots$ O clash is more important. Thus, the change in ϕ predominates, so that the β-sheet twist is right-handed, as can easily be understood by rotating the bonds in the ball–and–stick model. However, exceptions can occur locally if special values of the dihedral angles, χ, cause large side chains to be located nearer to an NH group than to a CO group.

The overall effect is reflected in the Ramachandran plot: the right-handed tendency induced by an increase in ϕ is, for most points, larger than the left-handed tendency due to a decrease in ψ. For a few points, the opposite twist of ψ leads to a local left-handed twist. Nevertheless, averaged over the entire β-sheet, the twist is usually right-handed (see also [3,24,31]).

The considerations via the ball–and–stick model enabled the understanding of the different roles of ϕ and ψ. Figure 6A,B show that ϕ controls the distances between the oxygen and the subsequent side chain, as well as between the amino hydrogen and the side chain of the same amino acid. The increase in one distance results in the shortening of the other. The same also holds for ψ, governing the distances between the amino hydrogen atom and the previous amino acid, as well as the oxygen atom and the side chain of the same amino acid.

In teaching biochemistry, ball–and–stick models are well-suited because they allow a haptic perception. Computer models are quantitatively more exact but less memorable. Moreover, the latter models are usually visualized by a projection onto two dimensions, while ball–and–stick models retain the three-dimensional structure, which significantly improves the understanding of the impact of dihedral angles. For example, the effect of $C_{\beta }$… O clashes or of the bifurcated hydrogen bonds can be understood easily by using ball–and–stick models.

### 2.4. Implications for Tertiary Structure

The twisting and bending significantly influence the tertiary structure [4,19,32] (Figure 1). In particular, the right-handedness is consistent with the tilted arrangement in β-barrels and TIM barrels [8]. The sheets on the front side are orientated diagonally from bottom left to top right (or vice versa), while the sheets on the back side are arranged from bottom right to top left. This configuration defines the right-tilted type of β-barrels [1]. If the twist were left-handed, the inverse arrangement would be observed.

In any case, if the sheets are arranged in a diagonal direction (rather than vertically or horizontally), they need to be twisted to form a closed barrel. This is important for the function of proteins involving β-barrels, such as porins and lipocalins [33]. It can be speculated that the diagonal arrangement improves the stability and rigidity because the covalent bonds in the protein backbone can then resist tensions both in vertical and horizontal directions. This is in analogy to garden hoses reinforced by textile fibers, which are inserted in a diagonal direction as well.

Another important tertiary structure is the β-propeller [34]. Each ’blade’ usually consists of four antiparallel β-strands, arranged such that the innermost strand sits nearly perpendicular to the outermost one. Thus, the approximate twist angle between two neighboring strands can be calculated to be 30°. For the β-propeller, it does not matter whether the twist is right- or left-handed.

In general, β-β-β and β-α-β structural motifs form right-handed spirals, with some exceptions being left-handed [35]. β-α-β motifs occur, for example, in TIM barrels and Rossmann folds. The right-handed β-twist supports the chirality of that characteristic topology [23,36]. The twist is also important in incompletely folded states [23].

The tertiary structure seems to further restrict ϕ and ψ to an overall right-handed twist in order to control the distance between side chains of neighboring strands while preserving hydrogen bonding [1,32,33].

## 3. Discussion

Here, we have revisited the causes of the β-sheet twist. We have highlighted a molecular explanation of this phenomenon and the handedness of the twist, based on steric hindrance between atoms in the backbone and amino acid residues in the same strand. While this reasoning is in line with some earlier ideas [3,15,37], it extends them with respect to the various steric hindrances. The dihedral angles ϕ and ψ take on opposing roles with a short distance of side chains to oxygen being both the cause for the rotation of ϕ and the limiting factor for ψ and a short distance of side chains to an amino hydrogen being the cause for the rotation of ψ and the limiting factor for ϕ. Thus, four different molecular clashes have to be considered, which form an antisymmetric structure. This gives rise to a clockwise twist of ϕ which is not completely compensated by a counter-clockwise twist of ψ, thus leading to the commonly observed right-handed twist.

Earlier explanations [3,14,15,23,25,38,39] were difficult to understand and did not sufficiently consider the roles of ψ and ϕ and the antisymmetric effects of the two different $C_{\beta }$… O steric hindrances and the two different $C_{\beta }$… NH hindrances. The latter were insufficiently considered at all in the literature so far and the former two were not sufficiently distinguished.

According to our simulations, the β-sheet twist and its handedness can be explained by intrastrand interactions. This does not exclude that it is superimposed by interstrand interactions. As mentioned in Section 1.2, the steric hindrance between residues of neighboring strands plays an important role, particularly in antiparallel β-sheets [14,20,25]. This additional effect is an interesting question for future studies. In particular, the difference between the twisted conformations in parallel and antiparallel β-sheets can be elucidated [3,14].

We have outlined the relation of the β-sheet twist with tertiary structures such as the β-barrel, TIM barrel, and β-propeller. The twist is important for the function of proteins with such structures. A perfectly planar β-sheet cannot form a β-barrel [1]. Even though the present paper focuses on the role of the twist, a change of the torsional angles also induces a bend within the downstream chain. The bend leads to a curvature of the β-sheet, which is especially needed in the formation of tertiary structures like the β-barrel [4].

When a β-sheet involves a large percentage of glycine, such as in silk fibroin, then less steric hindrance should be expected because of the very small side chain of glycine [14,40]. This applies to some transmembrane beta-barrel proteins, in which the average twist/bend angles indeed tend to be smaller than those of water-soluble proteins [4]. For an analogous reason, amino acids that are branched at the $C_{\beta }$ atom such as valine and isoleucine favor a stronger twist than amino acids that are unbranched at that carbon [22,23].

It would be interesting to perform studies for distinguishing the effects of achirality and of the smallness of the side chain (as in glycine). To that end, achiral amino acids with larger side chains may be suitable, such as dehydroamino acids involving a double bond between the $C_{\alpha }$ and $C_{\beta }$ atom [41]. However, such studies might be difficult because dehydroamino amino acids favour a $2_{7}$ helix rather than a β-sheet [42].

Here, we have presented some figures with photographs of plastic molecule models. For more exact and quantitative analyses, computer simulations are better suited. Nevertheless, we suggest using plastic models in teaching biochemistry and structural biology because of their high didactic value. They retain the three-dimensional structure and allow a haptic perception and manual rotation of bonds.

The above analysis is relevant for protein design and synthetic biology. When designing new proteins, the twist should be taken into account and could even be influenced by choosing appropriate amino acids.

## 4. Methods

### 4.1. Ramachandran Plot

Although recent studies distinguish between twisting and bending, their common cause is a change in the dihedral angles [4,11,16]. The handedness of the twist depends on the dihedral angles within the peptide backbone (Figure 2). The dihedral angle of the peptide bond ω can be ignored because it can only adopt the two values of 180° and 0° with the latter value occurring very rarely. The values of ϕ and ψ as resulting from the underlying interactions within a strand and between different strands are depicted in the Ramachandran plot (Figure 3C) [43]. Note that this plot implies periodic boundary conditions: 180° is equivalent to −180°. Moreover, we recall that the dihedral angles are defined such that they increase upon a rotation in the clockwise direction when looking from the N- to the C-terminus.

The region of the beta-sheet is subdivided into two subregions by the diagonal line connecting the points (ϕ=−180°, ψ=180°) and (ϕ=180°, ψ=−180°) (Figure 3C). The points lying exactly on that line do not contribute to any twist. A twist occurs locally if the sum of the two angles ϕ and ψ is unequal to 0° and multiples of 180°. Due to the above-mentioned definition of dihedral angles, the twist is left-handed (i.e., counter-clockwise) if −180°<ϕ+ψ<0° or 180°<ϕ+ψ<360°. It is right-handed (i.e., clockwise) if −360°<ϕ+ψ<−180° or 0°<ϕ+ψ<180°. Note that when the sum of the two angles is near 180° or −180°, the structure is not really a β-strand anymore, so that we need not consider these cases.

### 4.2. Using the Avogadro Software

To illustrate the potential steric hindrance, the chemical editor Avogadro was used [27]. We chose this software because it is free, open-source, easy-to-use, and intuitive. As one of our goals is to make suggestions for teaching, we consider Avogadro to be an appropriate choice. First, we tried to utilize the successor Avogadro 2, but the necessary functions were defective. That is why the figures were produced with the older version of Avogadro in the following application versions: Avogadro Version 1.2.0, Library Version 1.2.0, Open Babel Version 2.3.90, Qt Version 4.8.6.

First, tripeptides were built via an internal menu. Then the geometry was optimized with the “MMFF94” force field, which is in general designed for organic compounds, to receive the correct atom bond distances. After that, all the torsion angles of the main backbone chain (without hydrogen atoms) were set to 180° (trans). These atoms were then fixed and the geometry of the side chains was optimized again to calculate the optimal conformation in the trans position of the backbone. The standard parameters were used and the calculation ran both times until the result did not improve any further. It must be considered that the calculated conformations may represent local optima. For better visualization, the molecules were all aligned in the same way. For the atoms that are very close to each other and for which the distances could be optimized by changing the dihedral angle, the van der Waals radii were indicated.

### 4.3. Ball–and–Stick-Models

The ball–and–stick models were constructed with the ’ORBIT’ molecule model kit (Cochranes of Oxford Ltd., Shipton-under-Wychwood, UK) [30]. The elongated size of the bonds eased the rotation and supported the observable twist. According to the tool, the scale is 300 million to one.

A tripeptide was constructed following the standard color-coding of atoms and scaled bond lengths specified in the manufacturer’s instructions. According to the bond length table of the kit, the bonds to hydrogen atoms were set to 2 cm. Due to missing connections of length 2.5 cm, the CO double bond was also set to 2 cm while the remaining C bonds were set to 3.5 cm as stated in the bond length table.

Observable short distances between non-bonded atoms indicate possible steric hindrances which might lead to a rotation of the dihedral angles. The individual angles of ϕ and ψ can be changed through a rotation of the respective connected NH group and O atom.

## Data Availability

Dataset available on request from the authors.
